# The left–right reversed visual feedback of the hand affects multisensory interaction within peripersonal space

**DOI:** 10.3758/s13414-023-02788-0

**Published:** 2023-09-27

**Authors:** Daisuke Mine, Takuji Narumi

**Affiliations:** 1 Graduate School of Interdisciplinary Information Studies, The University of Tokyo, Tokyo, Japan; 2 Graduate School of Information Science and Technology, The University of Tokyo, Tokyo, Japan

**Keywords:** Crossmodal congruency effect, Multisensory interaction, Peripersonal space, Spatial representation, Body representation

## Abstract

The interaction between vision and touch, known as the crossmodal congruency effect, has been extensively investigated in several research studies. Recent studies have revealed that the crossmodal congruency effect involves body representations. However, it is unclear how bodily information (e.g., location, posture, motion) is linked to visual and tactile inputs. Three experiments were conducted to investigate this issue. In Experiment 1, participants performed a crossmodal congruency task in which both their hand appearance and the motor trajectories were left–right reversed. The results showed that the crossmodal congruency effect was not observed in the reversal condition, whereas participants showed significant crossmodal congruency in the control condition, in which there was no visual manipulation of the hand. In Experiments 2 and 3, where either the hand appearance or motor trajectory was left–right reversed individually, a significant crossmodal congruency effect was observed. This study demonstrated that visual manipulation of hand appearance and motor trajectories both affected the crossmodal congruency effect, although neither showed a dominant effect that solely altered the crossmodal congruency effect. The present results provide insights into the relationship between visual-tactile interactions and bodily information.

## Introduction

We perceive external spaces using multiple sensory inputs. Many studies have revealed the effects of sensory inputs on sensory information perception (e.g., detection, localization, identification; Shams et al., 2005; Stein et al., 1996; for a review, see Sathian & Ramachandran, 2020). In most situations, these interactions occur automatically and inevitably. Notably, each sensory input is initially coded in receptor-specific reference frames and processed in sensory-specific brain areas. Therefore, the mode of interaction between sensory inputs is not simply determined by a spatial relationship with regard to an absolute space-based coordinate; rather, it depends on how they are processed in the sensory-specific neural flow and transformed into a common reference frame. The interaction between visual and tactile inputs is typically observed in spaces near the body. To address the mechanisms of such visual-tactile interactions, which occur in the surroundings of the body, we focused on how visual and tactile inputs are connected to pieces of bodily information related to the location of body parts (i.e., posture) and body movements.

The space near the body is called the peripersonal space (PPS); it is where people interact with the environment through their body movements (Rizzolatti et al., 1997). Neuropsychological and behavioral data have shown that information is processed within the portion surrounding the body surface not solely based on unisensory inputs but through the integration and interaction of multisensory inputs. Neuropsychological evidence has demonstrated that multisensory neurons exist in the frontoparietal area, especially in the ventral premotor cortex (vPMC) and intraparietal sulcus (IPS); they have receptive fields for both tactile stimuli on specific body surfaces and visual or auditory stimuli around the tactile stimuli. Behavioral studies have shown that responses to tactile stimuli are influenced by task-irrelevant visual or auditory stimuli presented within PPS by using crossmodal congruency tasks (Spence et al., 2000; Spence, Pavani, & Driver, 2004a; Zampini et al., 2007; for a review, see Maravita et al., 2003). In this task, participants held their thumb finger in a lower position and their index finger in an upper position and were asked to discriminate the elevation of the tactile stimulus delivered either to the thumb or index finger while ignoring the visual (or auditory) distractor simultaneously presented either around the thumb or index finger. Participants typically showed a faster response to tactile stimuli when the visual (or auditory) distractor was presented in a position congruent to the tactile stimulus (i.e., at the same elevation at which the tactile stimulus was delivered) compared with when the visual (or auditory) distractor was in an incongruent position (at a different elevation compared with that at which the tactile stimulus was delivered). This effect of visual (or auditory) stimuli on the reaction to tactile stimuli (i.e., the crossmodal congruency effect) is stronger when visual (or auditory) distractors are presented near the tactile stimuli—that is, within the PPS—rather than far from it. As these visual-tactile interactions can be observed even when the hands are crossed across the body midline, this multisensory interaction seems to be involved in hand representation (Spence, Pavani, & Driver, 2004a).

Several studies have revealed the various mechanisms underlying the crossmodal congruency effect. The spatial response conflict between the tactile target and visual distractor presents one major explanation for this effect. According to this explanation, responses to tactile targets are obstructed by different (wrong) response tendencies that are elicited by visual distractors presented at the opposite elevation compared with that of a tactile stimulation on incongruent trials. In contrast, responses to tactile targets are facilitated by the same (correct) response tendencies elicited by visual distractors presented at the same elevation on congruent trials. Other factors that induce the crossmodal congruency effect have also been proposed (e.g., multisensory integration and hand-mediated binding). The combination of these factors provides a comprehensive account for most of the previous findings regarding the crossmodal congruency effect (Shore et al., 2006; Spence, Pavani, & Driver, 2004a). However, Marini et al. (2017) examined the contributions of each possible factor to the crossmodal congruency effect using a modified version of the crossmodal congruency task; they showed that the effects of multisensory integration and hand-mediated binding are relatively small compared with the spatial response conflict (see also Spence, Pavani, Maravita, et al., 2004).

How does the brain determine whether a pair of visual and tactile stimuli is congruent or incongruent? Complex systems support human spatial and body perceptions, as extensive sensory and motor information relevant to spatial and body perception is available; each chunk of information is processed differently in the brain (i.e., in different spatial coordinates). Several pieces of information related to the hand and surrounding space are necessary for determining the location of each sensory stimulus in relation to the body. The brain estimates the location of body parts and how the body moves. These estimations can be achieved by integrating visual and proprioceptive information regarding the hand as well as the relationship between these afferent signals and efferent motor commands. However, how each piece of information affects the crossmodal congruency effect remains unclear. Previous research (e.g., the work by Pavani et al., 2000) addressed how visual and proprioceptive information about the hand can contribute to the crossmodal congruency effect. In Pavani et al.’s (2000) study, participants performed a crossmodal congruency task while holding both hands below an opaque box to keep them concealed from view. Visual distractors were presented above the opaque box and not near the hands. This study demonstrated that the crossmodal congruency effect was stronger when artificial rubber hands were presented above the opaque box and near the visual distractors compared with the situation in which no rubber hands were presented. They interpreted this result to be a consequence of the multisensory integration of visual and proprioceptive cues for hand localization. Proprioceptive and tactile sensations were captured by the visually presented rubber hands, and the saliency of the visual distractors presented near the rubber hands increased. Pavani et al. (2000) demonstrated that visually presented hand representations that present information conflicting with proprioceptive information can affect the crossmodal congruency effect. However, it was difficult to characterize the effect of each piece of sensory information regarding the hand from the results of Pavani et al. (2000) because the relevant hand’s visual and proprioceptive cues were integrated into a common representation.

The present study focused on the mechanisms underlying the multisensory processes related to hand representations. To achieve this goal, we investigated the effect of the relevant hand’s left–right reversal visual feedback on the crossmodal congruency effect. In the present study, participants performed a modified version of the crossmodal congruency task after adapting to their hand’s left–right reversed visual feedback by using an immersive virtual reality technique. In the classic version of the crossmodal congruency task, participants must hold their thumb in a lower position and their index finger in an upper position. In the present study, the participants placed their right palm on the body midline with their thumb on the left side and their little finger on the right. The left–right reversal manipulation of the visual feedback of the hand leads to a conflict between the proprioceptive and visual cues of the hand posture (i.e., the location of each finger). When the tactile input was placed on the right thumb while keeping the palm down, the proprioceptive cue of the thumb location indicated the left side of the hand, whereas the visual cue of the thumb location was placed on the right side. Notably, visual and proprioceptive cues occupy almost the same spatial region, but cannot be integrated because they are mirrored. Furthermore, reversal manipulation flips the relationship between motor commands and their visual consequences. We conducted three experiments to examine how the hand posture’s visual appearance and the motor trajectory affected the crossmodal congruency effect.

## General methods

### Participants

We recruited 44 healthy participants as paid volunteers: 20 participants (mean age: 25.8 years; age range: 20–51 years) for Experiment 1, 12 participants (mean age: 23.0 years; age range: 20–27 years) for Experiment 2, and 12 participants (mean age: 23.4 years; age range: 20–37 years) for Experiment 3. We calculated the sample size for Experiment 1 based on a desired power of 0.95 and the assumed effect size (*f* = .35). The required sample size was 19; however, to counterbalance the order of the conditions, we recruited 20 participants for Experiment 1. The sample sizes for Experiments 2 and 3 were calculated from the result of the nonreversal condition in Experiment 1 (desired power: 0.95, effect size: *d* = 1.19). The required sample sizes for Experiment 2 and 3 were 12. All participants were right-handed and had normal or corrected-to-normal stereo vision. All participants provided written informed consent before participating in the experiments. The local ethics committee approved the experiments and procedures described below. The experiments were conducted in accordance with the principles and guidelines of the Declaration of Helsinki.

### Apparatus and stimuli

The participants viewed a virtual environment through a head-mounted display (HMD; (HTC VIVE Pro, displaying a stereoscopic image with a resolution of 2,880 × 1,600 and a field view of 110 °). In the virtual environment, a virtual right-hand avatar was moved synchronously or left–right reversed from each participant’s right-hand movements. The participant’s hand movements were tracked using an HTC VIVE tracker. A virtual world was developed using Unity3D and run on a Windows PC (Alienware M15 R4, Intel Core i7-10870H, 16 GB RAM, and NVIDIA GeForce RTX 3080). Vibrators (HAPTIC Reactor AFT14, ALPS ALPINE Co., Ltd.) were used to deliver tactile inputs to participant’s fingers.

## Experiment 1

### Procedure

**Fig. 1:**
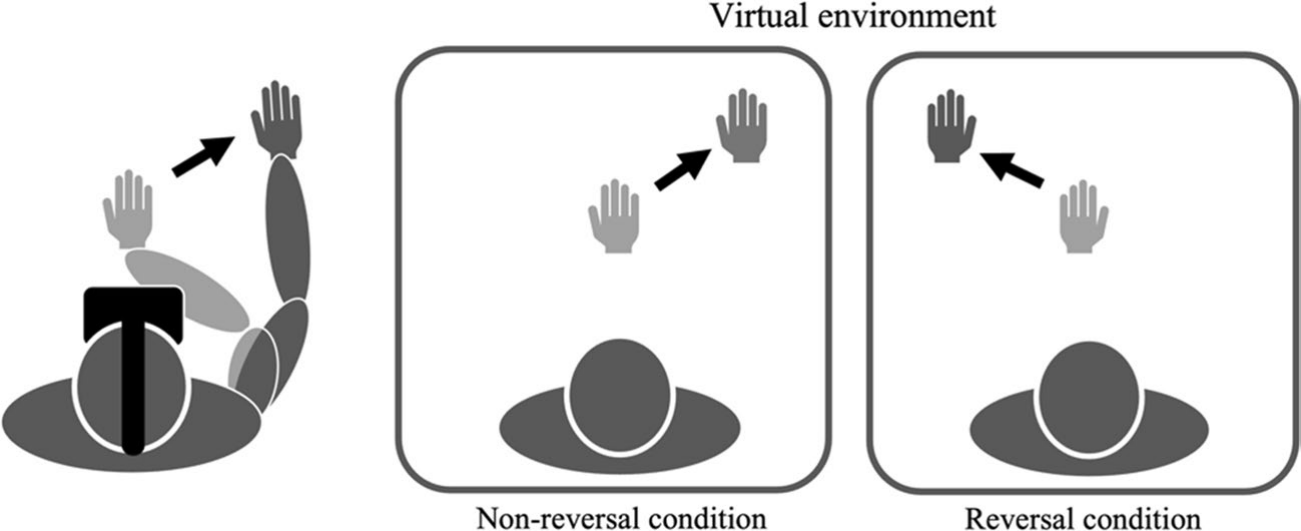
Experimental conditions in Experiment 1. Participants saw the virtual right-hand avatar whose appearance and motion were almost identical to the actual right hand’s appearance and motion in the nonreversal condition, while the motor trajectories were left–right reversed as the real right hand’s appearance and motion in the reversal condition

The experiment was conducted over 2 days. The participants performed two tasks—the adaptation task and the crossmodal congruency task—on each day. In these tasks, the virtual hand avatar’s movement was perfectly synchronized with participant’s right-hand movements (nonreversal condition) on one day and perfectly synchronized with the left–right reversed movements (reversal condition) on the other day (Fig. 1). Half of the participants were assigned to the nonreversal condition on the first day and to the reversal condition on the second day. In contrast, the other half was assigned to the reversal condition on the first day and the nonreversal condition on the second day.

**Fig. 2:**
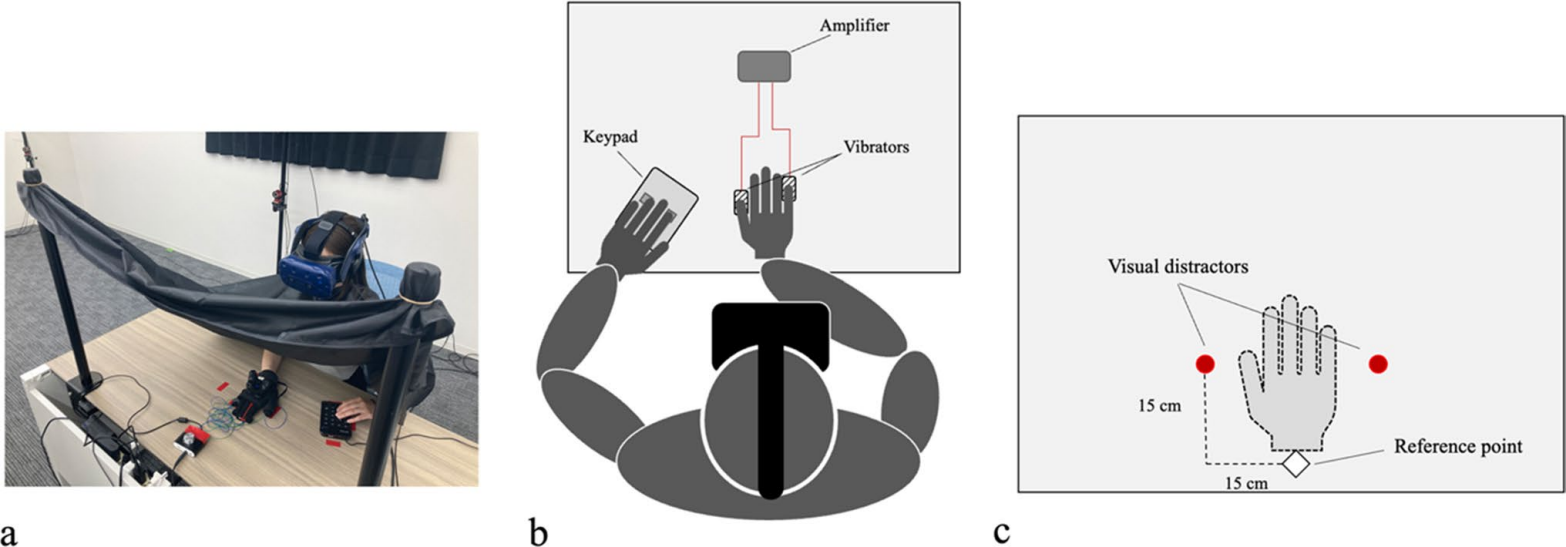
Experimental setup of the crossmodal congruency task. **a** Participants placed their hands below the barber’s cape to occlude the hands. **b** A schematic view of the experimental setup in the real environment. Participants reacted to tactile targets delivered from the vibrators attached to their thumb and little fingers by pressing the button with their left index and ring fingers. **c** A bird’s-eye view of the experimental setup in the virtual environment that participants viewed through the HMD. Participants placed the wrist of the virtual hand avatar on the reference point before each trial started. The visual distractor was presented on either the left or right side of the hand simultaneously with the tactile target. (Color figure online)

The participants sat at one end of a table In the lab space. Before the experiment started, the vibrators were attached to the participant’s right thumbs and little fingers, and a motion tracker was attached to the back of the hand. The participants were then instructed to adjust the fit of the HMD. A barber’s cape was wrapped around the participant’s necks, and one end of the cape was attached to poles positioned in front of the participants to prevent them from seeing their actual right hand when they detached the HMD during breaks between experimental blocks (Fig. 2a). In the virtual environment, while a virtual right-hand avatar was presented in a position corresponding to the participant’s actual right hand in the nonreversal condition, a left–right reversed right-hand avatar (identical to a left-hand avatar) was presented symmetrically with respect to the sagittal plane regarding the position of the participant’s actual right hand in the reversal condition. First, the participants performed an adaptation task to learn the relationship between motor commands and visual feedback. The target blue cube was presented at a random degree from −50 degrees to 50 degrees (At 0 degrees, the cube was presented on the ﻿sagittal plane to the participants) along the azimuth of a circle with a radius of 20 cm around a reference point (a white cube on the edge of the virtual desk). Before each trial, the participants were instructed to place the wrist of the hand avatar on the reference point with their palms down. When the target cube appeared, the participants began to reach out toward it as accurately as possible with their right hand. When the hand avatar touched the cube, it disappeared. After the cube disappeared, the participants placed the hand avatar on the reference point again, and the next trial started. The adaptation task consisted of three blocks of 100 trials each. On average, the participants took almost 45 minutes to complete the adaptation task.

Second, the participants performed a crossmodal congruency task (Fig. 2b–c). The participants performed one trial of the adaptation task before each trial to prevent the effects of adaptation from disappearing. The participants reached the target block with the hand avatar and then withdrew the hand avatar to the reference point. After placing the avatar at the reference point, white noise was played from the headphones. White noise was used to mask the sound generated by activating the vibrators. After pseudorandomized intervals (from 1,000 ms to 1,400 ms) from the beginning of the white noise, a tactile stimulus was delivered to either the thumb or the little finger of the participant’s right hand. Participants were asked to discriminate the location to which the tactile target was delivered while ignoring a visual distractor presented on either side of the hand at the same time as the tactile stimulation. The tactile targets included three 50-ms bursts with a 50-ms interval between each burst. The visual distractors were virtual red lights, located vertically at 15 cm and horizontally at 15 cm or −15 cm from the reference point. When the tactile stimulus was delivered to the thumb, half of the participants were instructed to press the lower button with their left index finger and the upper button with their left ring finger when the tactile stimulus was delivered to the little finger, as quickly as possible. The other half pressed the upper button with their left ring finger when the tactile stimulus was delivered to the thumb and pressed the lower button with their left index finger when the tactile stimulus was delivered to the little finger.

After the participants pressed the button, the screen turned dark, and the next trial started. In this task, the participants performed 120 trials: two tactile stimulus positions [thumb or little] × two visual stimulus positions [left or right] × 24 repetitions and 24 catch trials (no tactile stimuli). During the experiment, the participants kept their palms down on the body midline—that is, their actual thumbs were placed on the left side of the body midline, and their little fingers were placed on the right side. Therefore, the pair of tactile stimuli on the thumb and visual stimulus on the left side or the tactile stimulus on the little finger and visual stimulus on the right side were located on the same side in the external coordinates. Trials in which visual and tactile stimuli were presented on the same side in the external space were called congruent trials, and trials in which visual and tactile stimuli were presented on different sides were called incongruent trials.

### Results and discussion

**Fig. 3:**
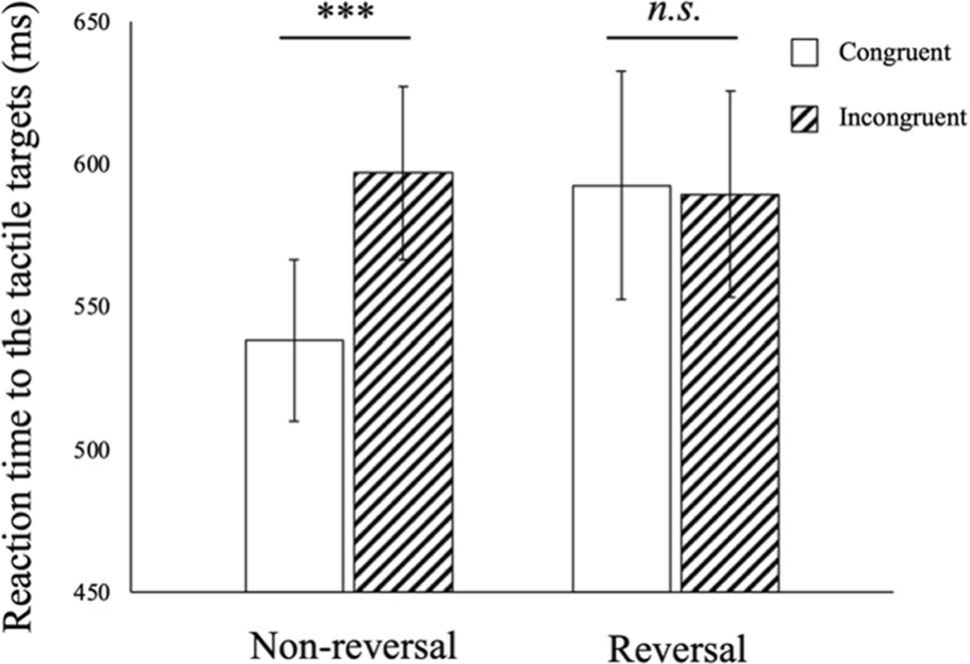
Reaction time to the tactile targets in Experiment 1. Error bars indicate the standard error. ***p < .001, and *n.s. p* > .05

To compare the effect of visual feedback modulation (nonreversal vs. reversal) on reaction time to tactile stimuli, a two-way analysis of variance (ANOVA) was conducted on reaction time to tactile stimuli in all the conditions. Trials with an incorrect response were removed from the reaction time analyses (6.3% of the trials). Outliers were defined as reaction times that exceeded 1,500 ms or 2.5 times the standard deviation from each participant’s mean reaction time for each condition; these were also excluded from the following analyses (2.0% of the trials). All the relevant analyses were conducted using the statistical software package R. Figure 3 shows the reaction time to the congruent stimuli (the visual and tactile stimuli are on the same spatial side) and incongruent stimuli (the visual and tactile stimuli are on different spatial sides) in each visual feedback condition.

A two-way repeated-measures ANOVA revealed that a significant main effect of congruency was observed, *F*(1, 19) = 17.75, *p* < .001, *η*_*p*_ = .483. Furthermore, an interaction effect between visual feedback modulation and congruency was observed, *F*(1, 19) = 10.38, *p* = .0045, *η*_*p*_ = .353. The main effect of visual feedback modulation was not significant, *F*(1, 19) = 1.51, *p* = .234, *η*_*p*_ = .074.

To interpret the source of the interaction effect between congruency and visual feedback modulation, we conducted post hoc Bonferroni-corrected *t* tests. In the nonreversal condition, a significant congruency effect was observed, *t*(19) = 7.08, *p* < .001, suggesting that the spatial congruency between the visual distractors and tactile targets affected the response time to the tactile stimuli. In contrast, no significant congruency effect was observed in the reversal condition, *t*(19) = 0.21, *p* = .834, suggesting that the presentation of the visual stimuli did not differently influence the response time to the spatially congruent and incongruent tactile stimuli.

A similar analysis was conducted on the error data. The two-way ANOVA revealed no significant main effects or an interaction effect—main effect of congruency: *F*(1, 19) = 3.80, *p* = .066, *η*_*p*_ = .010; main effect of visual feedback modulation: *F*(1, 19) = 0.18, *p* = .674, *η*_*p*_ = .167; interaction effect: *F*(1, 19) = 3.16, *p* = .092, *η*_*p*_ = .143. In previous studies, the crossmodal congruency effect was also observed in error data, as was observed in reaction times (Spence, Pavani, & Driver, 2004a). In the present study, error rates were generally low in all conditions (the highest error rate was observed in the incongruent trials in the nonreversal condition (*M* = 6.7%)). This may explain why the crossmodal congruency effect was not observed in the error data.

Experiment 1 aimed to evaluate whether visual left–right reversal manipulation on the right side affected the crossmodal congruency effect. The results showed that congruency affected the tactile response in the nonreversal condition but not in the reversal condition. In the nonreversal condition, a significant difference in reaction time was observed between congruent and incongruent trials, as shown in previous studies (e.g., Spence, Pavani, & Driver, 2004a). In contrast, no interaction effect was observed in either the congruent or incongruent trials in the reversal condition.

As part of the exploratory analysis, we examined two possible explanations for the disappearance of the crossmodal congruency effect in the reversal condition. First, the congruency between the visual distractors and tactile targets had no effect on tactile localization in this condition. Second, the effect of the visual distractor occurred; however, its direction differed across individuals. If the proprioceptive information of the hand had played a dominant role in judging the location of the sensory stimuli, a visual distractor on the same side should have facilitated tactile judgment, and a visual distractor on a different side should have obstructed it. However, if the visual information of the hand did have a dominant role, visual distractors affected tactile judgment in the opposite direction. If individual differences in the reliability of these two modalities countervailed each other, the crossmodal congruency effect would not be consistently observed. To discriminate between these two possibilities, we conducted a paired *t* test on the absolute value of the difference in reaction times between congruent and incongruent trials. The *t* test showed a nearly significant difference between the reversal (*M* = 40.8 ms) and nonreversal (*M* = 60.1 ms) conditions, *t*(19) = 2.09, *p* = .050. Certainly, an absence of statistical significance cannot lead to any concrete conclusion. Future studies need to further investigate these two possibilities, namely individual differences in the direction of the effect or general disappearance of the effect. However, based on the nearly significant result, we speculate that the congruency between the visual distractors and tactile targets generally had no effect on tactile localization in the reversed condition.

These results support the idea that the visual information of the left–right reversed hand affects the judgment of the tactile target location. However, the left–right reversed visual feedback in Experiment 1 manipulated both the visual feedback of the hand appearance (i.e., the position of the fingers relative to the hand) and the motion trajectory. Two additional experiments were conducted to investigate the individual effects of these factors. In Experiment 2, the appearance of the hand was left–right reversed, but the motor trajectory remained normal. In contrast, in Experiment 3, the motion trajectory was left–right reversed, but the appearance of the hand remained normal.

## Experiments 2 and 3

### Procedure

**Fig. 4:**
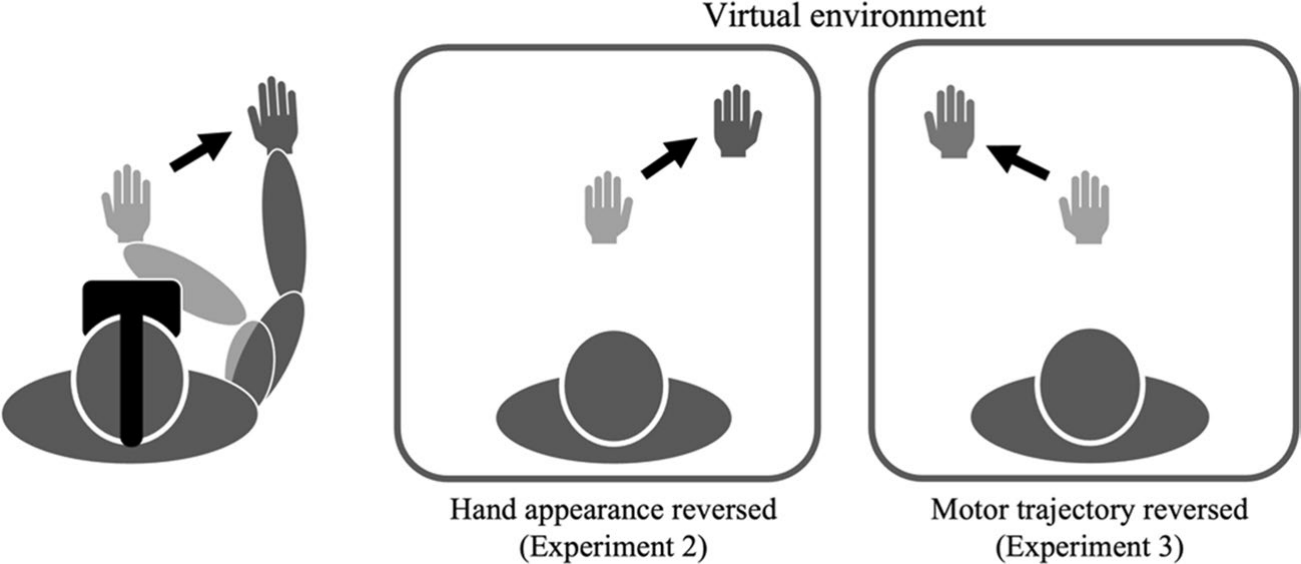
Experimental conditions in Experiments 2 and 3

Twelve paid volunteers participated in each experiment and performed the adaptation and crossmodal congruency tasks, which followed almost the same procedures as those outlined in Experiment 1, with the following two exceptions: first, the visual feedback of the hand appearance was left–right reversed, but the motor trajectory was not manipulated in Experiment 2; and the visual feedback of the motor trajectory was left–right reversed, but the appearance of the hand was not manipulated in Experiment 3 (Fig. 4). Second, although Experiment 1 was split over 2 days (assigning the normal visual feedback condition and the modulated visual feedback condition to each day, respectively), Experiments 2 and 3 used only the modulated visual feedback condition described above.

After the adaptation task consisting of three blocks of 100 trials, participants performed 120 trials of the crossmodal congruency task: two tactile stimulus positions [thumb or little] × two visual stimulus positions [left or right] × 24 repetitions, and 24 catch trials (no tactile stimuli). Trials in which visual and tactile stimuli were presented on the same side in the external space were called congruent trials, and trials in which visual and tactile stimuli were presented on different sides were called incongruent trials.

### Results and discussion

**Fig. 5:**
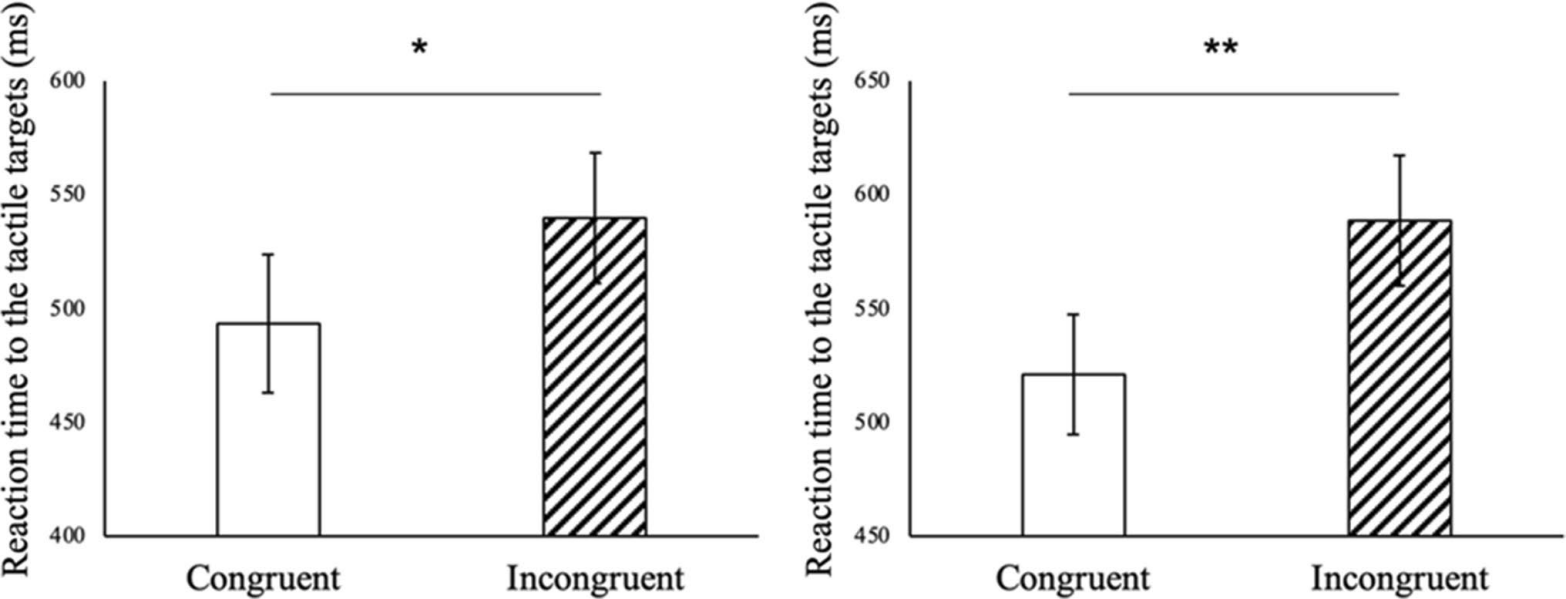
Reaction time to the tactile targets in Experiments 2 and 3. Error bars indicate the standard error. *p < .05, and **p < .01

To examine the presence of the congruency effect, paired *t* tests were conducted on the reaction times to tactile stimuli. Trials with an incorrect response were removed from the reaction time analyses (3.9% and 5.7% of the trials in Experiments 2 and 3, respectively). Outliers were defined as reaction times that exceeded 1,500 ms or 2.5 times the standard deviation from each participant’s mean reaction time for each condition; these were also excluded from the following analyses (1.6 % and 0.9 % of the trials in Experiments 2 and 3, respectively). Moreover, because one participant’s data in Experiment 3 could not be correctly recorded owing to machinery errors, that participant’s data were also excluded from the analyses. All analyses described below were conducted using the statistical software package R. Figure 5 shows the reaction time to the congruent stimuli (the visual and tactile stimuli on the same spatial side) and incongruent stimuli (the visual and tactile stimuli on different spatial sides) in each experiment.

Paired *t* tests revealed that, in both experiments, participants showed faster responses to the tactile stimuli when visual stimuli were presented in a spatially congruent position compared with when they were presented in an incongruent position—Experiment 2: *t*(11) = 3.05, *p* = .011; Experiment 3: *t*(10) = 4.48, *p* = .0012. These results demonstrated that the congruency effect between the visual and tactile inputs remained even when either the hand’s appearance or the motion trajectory was left–right reversed.

Paired *t* tests on the error data did not reveal significant differences in both experiments—Experiment 2: *t*(11) = 1.34, *p* = .206; Experiment 3: *t*(10) = 2.19, *p* = .053. Error rates were also low in these experiments, as in Experiment 1 (the highest error rate was observed in the incongruent trials in both experiments—Experiment 2: *M* = 4.8%; Experiment 3: *M* = 7.8%).

## General discussion

This study aimed to identify and investigate the aspects of sensory and motor information of the hand that affect the crossmodal congruency effect. In Experiment 1, all the visual information of each participant’s right hand was left–right reversed in the virtual environment—that is, we manipulated both the appearance of the hand itself and its motion trajectory. In this situation, the participants did not show faster responses on both the spatially congruent and incongruent trials. In comparison, they showed faster responses on the congruent trials compared with the incongruent trials when there was no manipulation of the hand’s visual feedback. In Experiments 2 and 3, we manipulated either the hand’s appearance or its motion trajectory. In both experiments, the participants reacted faster to tactile stimuli when spatially congruent visual stimuli were presented compared with when spatially incongruent visual stimuli were presented. Our results demonstrate that the visual manipulation of hand posture and motor trajectory both affected the crossmodal congruency effect, although neither had a dominant effect that solely altered the crossmodal congruency effect.

Recent works have shown the major role of spatial response conflict between the tactile target and visual distractor in the crossmodal congruency effect (Marini et al., 2017; Spence, Pavani, Maravita, & Holmes, 2004b). The response conflict account explains the crossmodal congruency effect as a conflict between response tendencies elicited by each sensory input. It should be noted that this account does not require the integration of visual and tactile inputs. Each tactile target and visual distractor may be processed depending on different spatial and bodily information—perhaps even in different reference frames. Spatial localization of tactile inputs involves the localization of tactile inputs on the body surface and the spatial localization—including posture—of the body parts (Longo et al., 2010; Tamè et al., 2019). The location and posture of body parts—especially that of the hand—reflect a visual cue in addition to the proprioceptive cue (Botvinick & Cohen, 1998; Makin et al., 2008). Many studies have used this visual capture effect to demonstrate that the visually induced perception of hand location and posture affects the processing of tactile inputs on the hand (de Vignemont et al., 2005; Pavani et al., 2000; Shore et al., 2005). However, the visual capture of the hand location and its effect on tactile processing are not observed when the artificial hand is placed in a location or posture that is implausible for the participants—that is, visual and proprioceptive cues cannot be integrated (Pavani et al., 2000). In this study, visual and proprioceptive cues of the hand posture (the azimuthal location of each finger; thumb on the left or little finger on the left) conflicted with each other in conditions involving reversed hand appearance (i.e., Experiments 1 and 2). The reverse manipulation of hand appearance resulted in a large dissociation between the visual and proprioceptive cues, suggesting that they could not be integrated. Thus, the estimation of hand posture and localization of the tactile targets were not influenced by the visual appearance of the hand when the hand appearance was reversed. Recent studies suggesting a dominant role of proprioception in tactile localization also support this idea (Liu & Medina, 2021). It is supposed that the tactile target is not mislocalized; rather, the visual distractor location in relation to the hand interferes with (i.e., delays or facilitates) judgment of the tactile location. Therefore, we could interpret that visual distractors were localized differently in the reversal condition in Experiment 1 compared with the other conditions in the present study.

Graziano (1999) investigated the contribution of visual and proprioceptive information from the hand to the visual reference frame (RF) of multisensory neurons in the premotor cortex of macaque monkeys. The visual RF of these neurons represented the space surrounding the hands of the monkey. Graziano (1999) demonstrated that these neurons responded to the location of a visually presented artificial hand when the actual hand was occluded. A more recent study on human subjects showed that the visual RF of neurons in the posterior IPS and lateral occipital complex (LOC) represents the space surrounding the visually presented hand and is independent of the proprioceptive information of the hand location (Makin et al., 2007). As these previous studies have suggested, specific areas of the brain process visual inputs that occur in the space around the visually presented hand. However, Makin et al. (2007) also showed that the neurons in the anterior IPS respond to the visual space surrounding the proprioceptive location of the hand. Therefore, the brain concurrently contains the visual spaces surrounding the visually perceived and proprioceptively perceived hands. Both vision- and proprioception-based maps representing the visual information surrounding the hand may contribute to identifying the location of visual distractors in relation to the hand.

A possible explanation for the effect of modulated motor trajectory on the crossmodal congruency effect is that visual distractors may be processed as potential action (reaching) targets. Molto et al. (2020) conducted a meta-analysis on the effect of action constraint on spatial perception and showed that the action constraint effect still occurs without motion intention. This result supports the idea that spatial properties automatically potentiate relevant actions (e.g., a small distance potentiates the reaching action). Moreover, the PPS representation, or its boundary, is tightly linked with the agent’s action capability, including having tools (Bassolino et al., 2010; Canzoneri et al., 2013; Iriki et al., 1996; Longo & Lourenco, 2006; Serino et al., 2007), and using a virtual body (Mine & Yokosawa, 2021, 2022), meaning that sensory inputs surrounding the body are automatically coded into a spatial map for action. Assuming reaching for a visual distractor, participants will have to move their arm to the opposite side compared with the location of the visual distractor when the motor trajectory is left–right reversed. Therefore, a visual distractor in the incongruent trials was located on the same (congruent) side as that of a tactile target in a spatial map for action, and vice versa in the congruent trials. If the action map contributes to determining the location of sensory inputs, it would reduce the crossmodal congruency effect in conditions with a reversed motor trajectory.

As discussed previously, vision-, proprioception-, and motion-based coding of visual distractors related to the hand may affect the judgment of tactile targets. However, because the reversed feedback on hand appearance and motor trajectory did not solely affect the crossmodal congruency effect (Experiments 2 and 3), the reversed visual or motor information of the hand had a relatively low influence on the localization of visual distractors. This is probably because reversed feedback of hand appearance and motor trajectory is relatively unreliable owing to fewer experiences in daily life as opposed to lifelong experiences with ordinal (nonreversed) hand appearance and motor trajectory. Hence, the visual distractors were processed in relation to the hand posture that is estimated proprioceptively. The reliability of each piece of information may help interpret the results of the reversal condition in Experiment 1, demonstrating that the crossmodal congruency effect disappeared and was not reversed (i.e., participants did not show tactile facilitation in incongruent trials or tactile obstruction in congruent trials). Under this condition, both the appearance and motor trajectory of the hand were reversed. Due to the contamination of these unreliable reversed cues, which conflicted with the proprioceptive hand posture, the localization of the visual distractors related to hand posture became uncertain. Thus, the effect of congruency between visual distractors and tactile targets disappeared.

In conclusion, we demonstrated evidence for the notion that visual and tactile inputs presented on or around the hand are not processed on the basis of a common representation of hand posture but through different sensory cues of hand posture when these cues conflict with each other and cannot be integrated. The location of the visual input might be parallelly processed in multiple reference frames independent of tactile processing and then integrated into a common spatial representation that interferes with the judgment of tactile localization. We also suggest the possibility that manipulating the motor trajectory affected the crossmodal congruency effect even though the participants kept their hands static when the visual and tactile stimuli were presented. However, the present study examined only the left–right reversed manipulation. People are relatively familiar with this manipulation because they see their bodies and motions through mirrors on a daily basis. Using other manipulations (e.g., rotation) may reveal different effects of hand appearance and motor trajectory on the crossmodal congruency effect. Moreover, manipulations of hand appearance and motor trajectory affect the embodiment of the hand. While we did not measure embodiment in the present study, many previous studies have reported that embodiment and multisensory perception affect each other (e.g., Blanke et al., 2015; Ehrsson, 2020; Tosi et al., 2023); thus, the embodiment of the hand can be a possible factor acting on the crossmodal congruency effect (also discussed in Spence, Pavani, Maravita, et al., 2004). Future studies should address these issues. Finally, the training in visuo-motor modulation was relatively short term (almost 45 minutes) because it would impose tremendous strain on participants to prevent them from seeing their actual hands for a long time. However, Werner and Bock (2010) suggested that adaptation to a left–right reversal is achieved more slowly than to other visuomotor distortions, such as a 180° rotation. Hence, it can be possible that a longer adaptation to the left–right reversed visual feedback affects the crossmodal congruency effect more drastically than we demonstrated. The important finding in our present study was that the modulation of the hand’s visual appearance and motor trajectory counteracted the existing crossmodal effect of the multisensory stimuli.

## Data Availability

The datasets generated during and/or analyzed during the present study are available from the corresponding author upon reasonable request.
